# The Voluntary Spirit v. State Control

**Published:** 1919-07-26

**Authors:** 


### GUILDS OF SERVICE.

### The Voluntary Spirit v. State Control.

THE Needlework and Linen Guilds connected with King Edward VII. Hospital, Cardiff, have held their annual meeting in the Thomas Andrews' Ward on the afternoon of the 2nd inst., and proved to be one of the most remarkable of a long series of such meetings. Lady Courtis, the president of the Linen Guild, expressed the feeling of everyone present in the preface to her report. She said : "We have met in this building to-day under the shadow of a great sorrow. Colonel Bruce Vaughan, who I think, I am right in saying was the greatest friend the hospital has ever had, has been called to his rest and we are all the poorer for his loss. He was a man who gave his life to the work of providing for the alleviation of suffering. We cannot walk through this beautiful hospital without seeing on every side evidences of his guiding hand. No earthly honour fell to his share, but can we doubt that he has received the commendation of Him who said, 'Inasmuch as ye have done it unto one of the least of these my brethren, ye have done it unto me.'" The usual garden party was abandoned, the music of the band was stilled, but on no previous occasion had the associates gathered in greater number or with greater zeal in the service of their guilds. Major General Lee, who presided, thanked them for the great help and encouragement of their work, as revealed by the reports of the presidents, Lady Cory (Needlework Guild) and Lady Courtis (Linen Association). Lady Cory said she regarded the standard of gifts received as higher than in previous years, whilst Lady Courtis reported the receipt of 642 articles at a money value of £138 and subscriptions in money of £131.

In thanking the guilds on behalf of the Medical Board, Major Paterson, its chairman, gave a thoughtful appreciation of the varied and increasing services which the growing regard for public health imposes upon the voluntary hospitals and the tendency to State control which follows, and appealed for even keener perseverance in work such as that undertaken by the guilds, which represents the best traditions of the voluntary spirit. Sir William James Thomas thanked General Lee for presiding, and referred to him as the Father of the hospital.

The associates were entertained to tea by Lady Cory and Lady Courtis, and then made a tour of the recent extensions, which are now sufficiently developed to reveal the scope and soundness of Colonel Bruce Vaughan's aspirations for the hospital.

